# Land-Use History and Contemporary Management Inform an Ecological Reference Model for Longleaf Pine Woodland Understory Plant Communities

**DOI:** 10.1371/journal.pone.0086604

**Published:** 2014-01-23

**Authors:** Lars A. Brudvig, John L. Orrock, Ellen I. Damschen, Cathy D. Collins, Philip G. Hahn, W. Brett Mattingly, Joseph W. Veldman, Joan L. Walker

**Affiliations:** 1 Department of Plant Biology, Michigan State University, East Lansing, Michigan, United States of America; 2 Department of Zoology, University of Wisconsin, Madison, Wisconsin, United States of America; 3 Department of Biology, Colby College, Waterville, Maine, United States of America; 4 U.S. Forest Service Southern Research Station, Clemson, South Carolina, United States of America; DOE Pacific Northwest National Laboratory, United States of America

## Abstract

Ecological restoration is frequently guided by reference conditions describing a successfully restored ecosystem; however, the causes and magnitude of ecosystem degradation vary, making simple knowledge of reference conditions insufficient for prioritizing and guiding restoration. Ecological reference models provide further guidance by quantifying reference conditions, as well as conditions at degraded states that deviate from reference conditions. Many reference models remain qualitative, however, limiting their utility. We quantified and evaluated a reference model for southeastern U.S. longleaf pine woodland understory plant communities. We used regression trees to classify 232 longleaf pine woodland sites at three locations along the Atlantic coastal plain based on relationships between understory plant community composition, soils (which broadly structure these communities), and factors associated with understory degradation, including fire frequency, agricultural history, and tree basal area. To understand the spatial generality of this model, we classified all sites together and for each of three study locations separately. Both the regional and location-specific models produced quantifiable degradation gradients–i.e., progressive deviation from conditions at 38 reference sites, based on understory species composition, diversity and total cover, litter depth, and other attributes. Regionally, fire suppression was the most important degrading factor, followed by agricultural history, but at individual locations, agricultural history or tree basal area was most important. At one location, the influence of a degrading factor depended on soil attributes. We suggest that our regional model can help prioritize longleaf pine woodland restoration across our study region; however, due to substantial landscape-to-landscape variation, local management decisions should take into account additional factors (e.g., soil attributes). Our study demonstrates the utility of quantifying degraded states and provides a series of hypotheses for future experimental restoration work. More broadly, our work provides a framework for developing and evaluating reference models that incorporate multiple, interactive anthropogenic drivers of ecosystem degradation.

## Introduction

Ecological restoration efforts guided by a target range of reference conditions [1] often fail to achieve these targets [2]. In part, this may be because knowledge of reference conditions, while useful, is by itself insufficient for guiding or prioritizing restoration due to variation in degraded states (unrestored conditions that deviate from reference conditions). Human-modified landscapes support a range of degraded states, resulting from land-use legacies and variation in contemporary management [3], [4]. Thus, an important early step in the restoration of human-modified landscapes is the formalization of ecological reference models, which describe both reference conditions and the spectrum of degraded states that are common for a given ecosystem [3], [5]. Reference models have been formulated for many ecosystems [6]–[8]; however, these models are frequently qualitative and models of ecosystem degradation have rarely been quantitatively developed or evaluated [4].

Data-driven ecological reference models that incorporate both the consequences (e.g., altered species compositions) and causes of degradation (e.g., altered disturbance regimes) can promote a better understanding of degraded landscapes, help to prioritize restoration and management activities, and contribute toward the goal of tailoring restoration strategies to specific degraded states [3]–[5]. For example, in fire-maintained ecosystems, qualitative reference models simply predict increasing degradation with fire suppression [6]–[8], but do not detail the nature of this relationship, such as the rate at which degradation increases with fire suppression or whether thresholds exist where degradation increases abruptly with fire suppression. In contrast, a quantitative reference model could describe degradation based on thresholds in fire frequency, allowing for sites to be classified along this axis, and restoration planning and approaches could be tailored accordingly [4].

We suggest that a quantitative ecological reference model should have three features to be both ecologically relevant and useful to land managers. First, it would classify sites based on relevant ecological communities, which in many cases will be plants – the basis for many management and restoration decisions [9], [10]. Second, suspected drivers of ecosystem degradation would be incorporated into the classification by linking site conditions to factors associated with degradation [4], [5]. Together, these two steps would describe the range of degraded states and quantify how, in terms of degrading factors, they differ from each other and from reference conditions. Third, to make such a model applicable to restoration management, it would use data routinely available to land managers across sites spanning landscapes, the scale at which restoration planning and many management efforts typically operate [4], [5], [11]. We define landscape as “an area that is spatially heterogeneous in at least one factor of interest” [12] – in our case, degradation of sites across a location for which restoration or management might be coordinated. In this study, we incorporate these three features to develop a quantitative ecological reference model for longleaf pine (*Pinus palustris*) woodlands at three locations (i.e., landscapes) in the southeastern United States.

**Figure 1:**
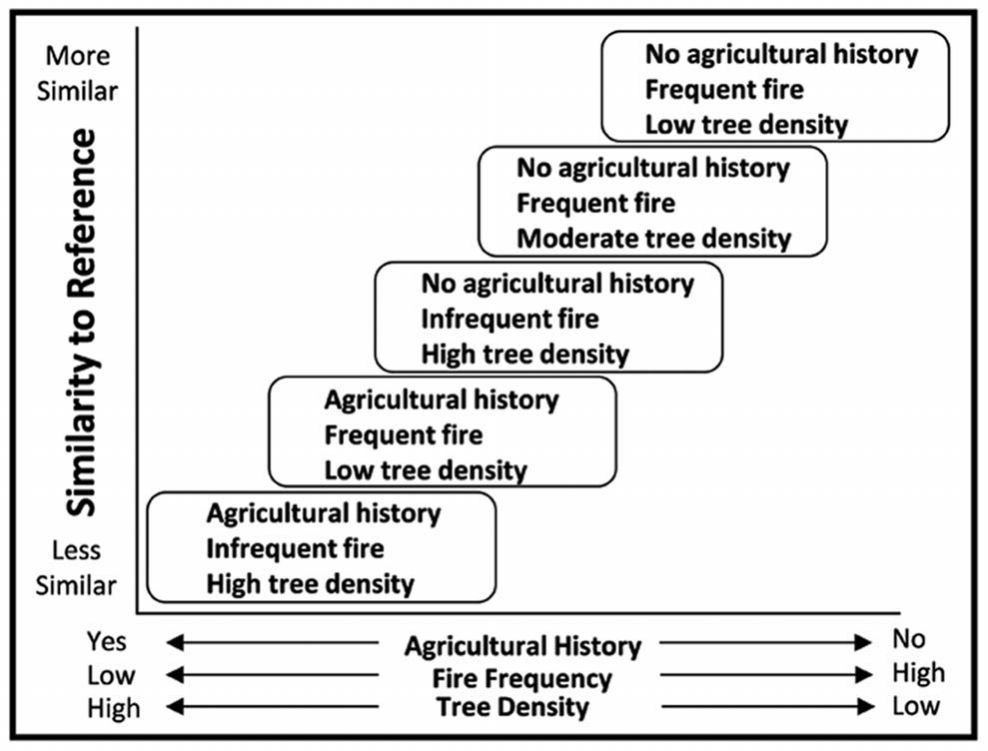
Conceptual model of degradation for understory plant communities in longleaf pine woodlands. Understory degradation (deviation in community composition from reference site conditions) is predicted to increase with occurrence of agricultural history, increasing overstory density, and declining fire frequency. Note: not all combinations of model components are presented in this figure and, while depicted as a linear process in this conceptual diagram, nonlinearities may exist during restoration from degraded states. Model is based on [8].

Fire-maintained longleaf pine woodlands support species-diverse understory plant communities that have been widely degraded by human land uses and are an active target for restoration [8]. The starting point for our work is a previously published qualitative reference model [8], which describes a degradation gradient in longleaf pine understory plant diversity and composition caused by past agricultural land use, altered fire regimes, and silvicultural activities (see also [13]). Agricultural legacies can persist for decades following abandonment, leading to reduced understory diversity and modified community composition on post-agricultural sites [14]–[16]. Fire suppression leads to increased tree abundance, canopy cover, and an accumulation of leaf litter and duff (i.e., forest floor), each of which may reduce understory diversity and modify understory community composition [17]–[20]. Historically, lightning and human-ignited surface fires burned longleaf pine woodlands as frequently as every 1–6 years, but today fire suppression is widespread and, where fires do occur, they are implemented through prescribed burning [21]. Overstory trees reduce understory plant diversity through competition with understory plants for light and water [18], [22]; tree density is also altered by silvicultural management, including tree planting and harvesting [8], [23]. Based on these consequences of human land use, we predict longleaf pine understory communities to be most degraded at sites with a history of agriculture, contemporary fire suppression, and a dense overstory (Figure 1), which are also determinants of degradation in many other ecosystems [24], [25]. Guided by this past work, we focus on fire history, agricultural legacies, and overstory density as likely degrading factors for longleaf pine understory communities; however, we know of no efforts to quantify a reference model based on these factors.

The goals of our study were to quantify and then evaluate a reference model for longleaf pine woodlands, based on a previously described qualitative model [8] and associated literature (references above). To achieve these goals, we pursued three specific objectives: 1) classify longleaf pine understory plant communities based on a set of previously identified degrading factors (agricultural history, fire frequency, overstory tree basal area; references above), 2) evaluate the spatial generality of this model by evaluating the roles of degrading factors across sites that vary in soil conditions and by comparing models for three different landscape-scale locations and a region-scale model (three locations combined), and 3) evaluate the resulting regional and location-specific reference models by comparing degraded states to a set of reference sites.

**Figure 2:**
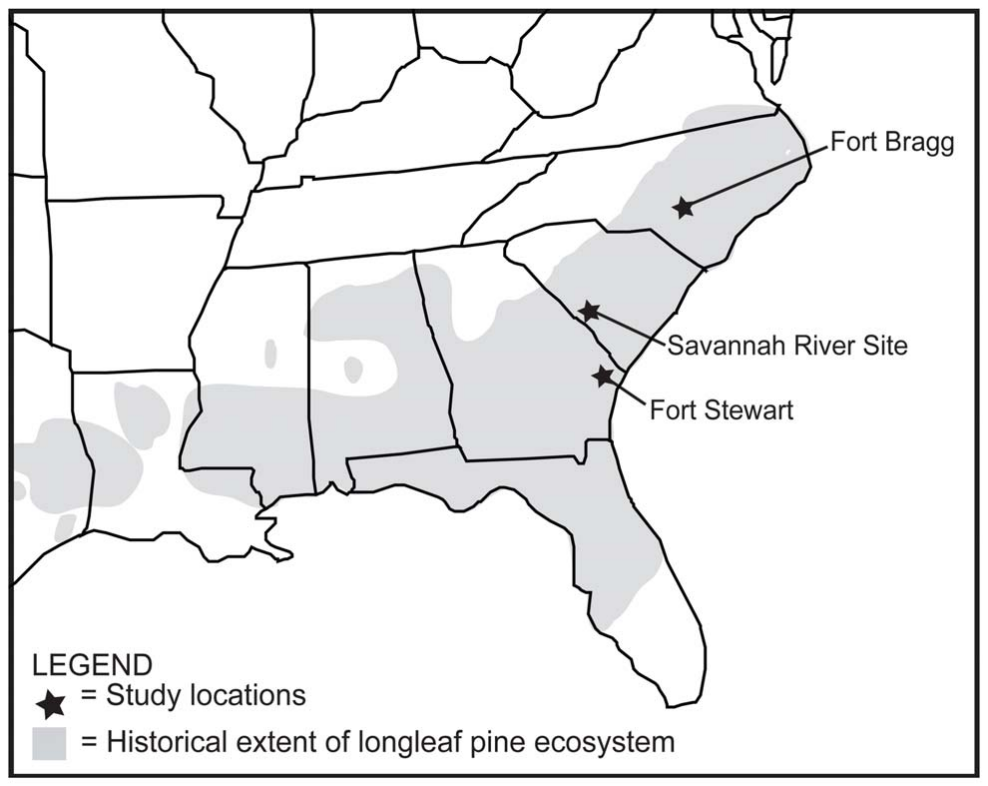
Geographic range of the longleaf pine ecosystem and map of study locations. Study locations (Fort Bragg [NC], 31°11' N, 79°15' W; Fort Stewart [GA], 31°56' N, 81°36' W; and Savannah River Site [SC], 33°20' N, 81°40' W) were in three different physiographic regions (sandhills, southern coastal plain, and Atlantic coastal plain, respectively [26]) allowing for creation and evaluation of an ecological reference model across a range of ecological settings. This model was based on data from 232 sites, which varied in their levels of degradation, with results subsequently compared to data from 38 reference sites.

We pursued our first objective through regression tree analysis and data from 232 longleaf pine woodland sites, which were selected to span a range of biophysical conditions across our three study locations in the southeastern U.S. (Figure 2). This analysis groups sites with similar plant communities based on data-defined levels and combinations of agricultural history, fire frequency, and overstory tree basal area. We also included soil attributes in this classification because of their importance for determining plant community composition in this system [26] and to assist with our second objective. We recognize that additional degrading factors might be identified (e.g., invasive species); however, we selected this set for consideration based on clear linkages with understory degradation (references above), applicability to our study landscapes (e.g., invasive species were in low abundance at all of our study sites), and the likelihood that these data would be easily obtained by land managers, facilitating application of the model to land management and restoration planning. We pursued our second objective in two ways. First, the inclusion of soil variables in our models provides insight into whether degrading factors (e.g., fire frequency) operate generally to determine degraded states, or differently for some types of longleaf pine woodlands compared to others (e.g., those that occur on Entisols vs. Ultisols). Second, by including three locations in our study, we can evaluate the generality of a regional model (spanning all sites) relative to models for three separate landscapes (Table 1). This is important for understanding the degree to which we can generalize our results across a region or to which landscape-to-landscape variation might preclude broad application of the regional model. Finally, to address our third objective, we compared the degraded states resulting from the regional and location-specific models to a set of 38 reference sites based on a suite of biophysical characteristics that are relevant to restoration and land management. In doing so, we evaluated whether each model produces a quantifiable gradient in degradation. Given the geographic distribution of our study locations, our results may be most applicable to Atlantic Coast longleaf pine woodlands, but the degrading factors we study – agricultural legacies, altered fire regimes, and altered overstory tree abundance – are broadly relevant across the longleaf pine ecosystem [8], [13].

**Table 1:** Attributes across study sites and for each of the three study locations.

Variable	All Sites	Fort Bragg	Fort Stewart	Savannah River Site
Number of sites	232	84	68	80
Canopy cover (%)	51.7±2.8	30.8±1.9	62.7±4.8	64.5±3.5
Total basal area (m^2^/ha)	18.8±1.0	19.4±1.5	15.8±1.9	20.7±1.8
Pinus basal area (m^2^/ha)	17.0±1.0	17.6±1.7	14.2±1.7	18.8±1.6
Non-Pinus basal area (m^2^/ha)	1.8±0.4	1.8±0.8	1.7±0.6	1.8±0.8
Years since fire	3.4±0.8	1.0±0.2	2.3±0.7	6.8±2.1
Number of fires (1991–2009)	4.6±0.3	5.8±0.3	5.0±0.6	3.0±0.5
Soil water holding capacity (%)	39.5±0.8	41.2±1.4	40.8±1.6	36.8±1.0
Soil organic matter	1.8±0.1	2.0±0.2	2.0±0.2	1.5±0.1
Species richness/m^2^	5.1±0.4	4.7±0.6	6.7±0.8	4.2±0.6
Species evenness/m^2^	0.7±0.02	0.6±0.03	0.7±0.02	0.7±0.02
Vegetation cover (%)	23.2±2.3	13.4±2.0	38.3±4.7	20.5±3.0
Bare ground (%)	8.2±1.5	11.4±2.7	10.2±3.2	3.1±1.2
Down woody debris (%)	5.2±0.7	2.1±0.4	5.2±1.2	8.4±1.5
Litter depth (cm)	2.2±0.3	1.0±0.1	2.7±0.4	3.1±0.4
Duff depth (cm)	0.9±0.2	0.2±0.03	0.6±0.2	1.9±0.3

Values exclude data from reference sites and are mean ±95% confidence interval.

## Methods

### Ethics Statement

Prior to conducting field data collection, we obtained approvals from the Fish and Wildlife Branch and Forestry Branch at Fort Stewart, the USDA Forest Service-Savannah River at Savannah River Site (a National Environmental Research Park), and the Endangered Species Branch, Forestry Branch, and the Cultural Resources Program at Fort Bragg. All data collection occurred on publicly owned land. We observed and surveyed, but made no collections of protected species.

### Study System

This study was carried out in fire-dependent longleaf pine woodlands on sandy soils of the southeastern United States (Figure 2). Frequently burned longleaf pine woodlands are characterized by a relatively sparse, longleaf pine-dominated overstory and a species-diverse understory of graminoids, forbs, and shrubs [21], [26]. Longleaf pine ecosystems, of which woodlands are a component, span from Texas to Virginia (Figure 2), but today less than 3% of historic area remains due to agricultural conversion, urbanization, and fire suppression [21]. As such, longleaf pine ecosystems are of high priority for conservation and restoration [8], [21].

We sampled longleaf pine woodlands at three locations within the historical range of the longleaf pine ecosystem: Fort Bragg (North Carolina), Fort Stewart (Georgia), and Savannah River Site (South Carolina) (Figure 2). Like much of the longleaf pine region [21], the landscapes at these study locations were historically fragmented by agriculture. Following federal government acquisition (Fort Bragg: 1919, Fort Stewart: 1940, Savannah River Site: 1951) agriculture was abandoned, resulting in contemporary landscapes supporting mosaics of longleaf pine woodlands with and without agricultural land-use histories. Study locations are managed with prescribed fire, but substantial variation in recent fire history exists across the sites, resulting in a range of fire histories from frequently burned to fire suppressed (Table 1).

### Site Selection

We selected a set of study sites that characterized the range in variation of degraded conditions (fire history, overstory density, agricultural history) at each location. Sites were each ≥1 ha, supported overstory longleaf pines, and lacked firebreaks, drainages, or other features causing abrupt transitions in understory vegetation. Sites varied in overstory tree density, recent fire history (1991–2009), and agricultural land-use history (Table 1); stand age from the 165 of 232 non-reference sites with available data was 62±1.2 years (mean±1SE). We classified each site as having a “forest” or “agricultural” land-use history based on its status in historical aerial photographs (Fort Stewart, Savannah River Site) or maps (Fort Bragg) from the year of federal acquisition. We obtained GIS data from prescribed fire managers at each location to reconstruct fire history (prescribed and wild) between 1991 and 2009 for each site. Sites were considered burned in a given year if they occurred within the boundaries of a fire management unit that was burned in that year. We determined overstory basal area for each site during vegetation sampling.

We quantified reference conditions by sampling a set of reference sites at each location (Fort Bragg n = 15, Fort Stewart n = 14, Savannah River Site n = 9). These sites had been previously identified by regional botanical experts at the Carolina Vegetation Survey (CVS) “to document the composition and status of the natural vegetation of the Carolinas” (http://cvs.bio.unc.edu/). Reference sites had no known history of cultivation, were generally well maintained by prescribed fire, and were located within the boundaries of the respective study locations.

All study sites were located on soils that are primary substrates for longleaf pine communities [26], with three soil orders characterizing the majority of our sites (97%): 212 of our 270 sites (78.5%) were located on Ultisols, 38 sites (14.0%) were on Entisols, 13 sites (4.8%) were on Spodosols, and 7 sites (2.6%) were on Inceptisols.

### Data Collection

Between 20 August and 13 November 2009, we surveyed the 270 study sites: 99 at Fort Bragg; 82 at Fort Stewart; 89 at Savannah River Site. We used one randomly located and oriented 20 m×50 m plot at each site. This plot design was a modified version of the CVS protocol [27], which is broadly employed throughout and beyond our region to characterize forest, savanna, and grassland plant communities. We identified and assigned a percent cover value to all understory plant species (herbaceous species and woody species <2.5 cm diameter at 1.4 m height) rooted within or overhanging each of eight 1 m×1 m subplots located within a 20 m×20 m portion of each plot. Taxonomy follows Radford and colleagues [28], except for the genus *Dichanthelium*, which follows Weakley [29], and the genera *Lyonia* and *Persea*, which follow Wunderlin and Hansen [30]. Within each subplot, we estimated the percent cover of green vegetation, bare ground, and down woody debris (logs, sticks, pine cones, and bark). We measured the depth of leaf litter and duff in the center of each subplot. To characterize overstory conditions within each 20 m×50 m plot, we recorded canopy cover with a spherical densiometer held at 1.4 m above six points spaced at 10 m intervals along the plot center line, and identified and measured the diameter of all trees ≥2.5 cm diameter at 1.4 m, within the plot.

To characterize site-level soil conditions, we collected soil cores (2.5 cm diameter by 15 cm deep) at 10 m intervals along the center line of each 20 m×50 m plot. Samples were composited by site and analyzed by Brookside Laboratories, Inc. (New Knoxville, OH) for soil organic matter content (SOM) [31], which is an indicator of soil degradation in post-agricultural forests [3], [24]. We also analyzed soil samples for water holding capacity as the proportionate difference between saturated wet weight and oven-dried weight, following the method of [32], as described by [15]. Soil moisture availability correlates with longleaf pine understory diversity and productivity [33].

### Statistical Methods

Following data collection, we constructed a site-by-species matrix for subsequent plant community analyses, using the mean cover for each species across the eight subplots at each site. To develop ecological reference models, we used multivariate classification and regression tree analysis to classify non-reference sites based on a combination of plant community composition and environmental data [34]. This analysis creates a dichotomous tree with splits based on environmental data that minimize compositional dissimilarity within groups of sites (i.e., ‘classes’). In our case, we used environmental data that corresponded to the degrading factors in Figure 1: agricultural history, tree basal area (total basal area, *Pinus* spp. basal area, non-*Pinus* spp. basal area), and fire frequency (number of burns 1991–2009, time since last fire). We investigated *Pinus* and non-*Pinus* overstory separately because these groups respond differently to fire frequency (*Pinus* is more fire tolerant; [35], [36]) and because land managers often remove hardwoods during restoration [8], [20]. We also included soil order (Entisol, Inceptisol, Spodosol, Ultisol) and soil water holding capacity in the classification, which are two aspects of soils that broadly structure longleaf pine communities [26]. As we explain above, the inclusion of these soil variables in our models provides an opportunity to evaluate the generality of degrading factors across longleaf pine communities that occur on different substrates. We conducted classifications for all 232 non-reference sites together to accomplish our first objective (‘All Sites’ analysis) and for each of the three locations separately to address our second objective (‘Fort Bragg’, ‘Fort Stewart’, and ‘Savannah River Site’ analyses). To determine the number of final classes generated by each analysis, we conducted 500 cross-validations of the model and selected the most frequently occurring tree size using the 1-SE rule [37]. For ease of interpretation, we numbered the resulting classes to align with Figure 1, so that Class 1 was most degraded. Classes defined by soil attributes do not align with our conceptual framework in Figure 1 and we do not attempt to attribute degradation to soil conditions; however, we attempt to integrate soil-defined classes into this alignment based on divergence in biophysical factors from reference conditions (see below).

To accomplish our third objective, we evaluated the ecological relevance of the classes resulting from our classification analyses by comparing biophysical attributes of classes to reference sites. We calculated means per site (±95% CI) of understory richness and evenness, canopy cover, basal area (total, *Pinus*, non-*Pinus*), years since last fire, number of fires between 1991 and 2009, percent cover of vegetation, bare ground, and down woody debris, litter and duff depth, SOM, and soil water holding capacity. To visualize plant community composition among classes and to compare these classes to reference sites, we used Canonical Analysis of Principal Coordinates (CAP), with Bray-Curtis similarity as the distance measure following square-root transformation of the raw species abundance data [38]. CAP is a constrained ordination analysis that characterizes multivariate differences among groups (i.e., classes, reference sites) [38]. We note that, while many of the attributes we investigated were unrelated to our classification analyses, some attributes were components of the classifications (e.g., community composition). The purpose of these analyses was to quantitatively compare among classes and reference sites, rather than to formally test research hypotheses. To identify species that distinguished classes and reference sites from one another, we used Indicator Species Analysis (ISA) [39]. ISA results are presented in Tables S1, S2, S3 and S4. We ran separate analyses for each of the four classifications (All Sites, Fort Bragg, Fort Stewart, Savannah River Site). We used PRIMER-E version 6 for CAP analyses and calculation of species richness and Pielou’s evenness [40], SAS version 9.2 for calculation of confidence intervals [41], R version 3.0 for multivariate regression trees [42], and PC-ORD version 5.31 for ISA [43].

## Results

### All Sites Classification

**Figure 3:**
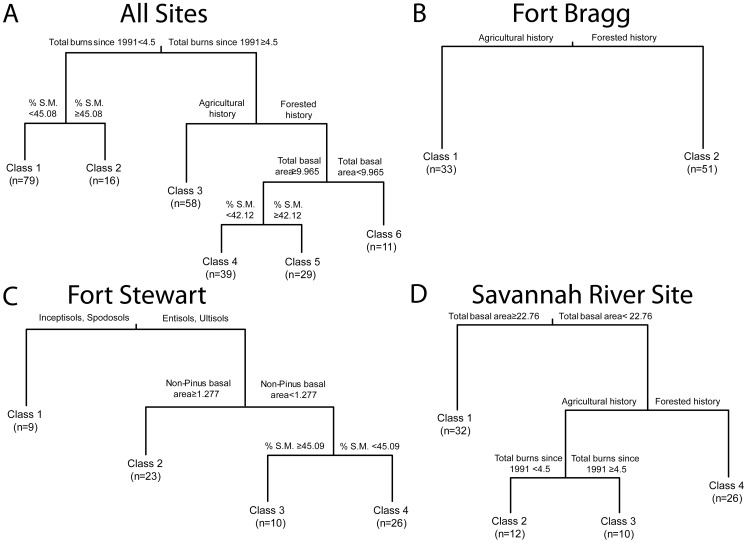
Results of multivariate classification and regression tree analysis. A) All study sites, B-D) separate study locations. In each analysis, sites are classified based on plant community composition and environmental data and classes are ordered to align with the conceptual model in Figure 1 (Class 1 =  most degraded), with the exception of Class 1 at Fort Stewart, which represents a soil-vegetation association not related to degradation. Branch length at each split is scaled to the variance explained by the corresponding environmental factor. The number of study sites in each class is presented below each class label.

The classification of All Sites from the combined three locations resulted in five splits and six classes (Figure 3a). Sites first split according to fire frequency. Infrequently burned sites (≤4 burns since 1991) were further classified based on soil moisture: Class 1 was characterized by infrequent fire and lower soil moisture (<45.08%), whereas Class 2 was characterized by infrequent fire and higher soil moisture (≥45.08%). Frequently burned sites were further classified based on land-use history, with Class 3 characterized by frequent fire and agricultural history. Among sites with forested history, the model made two splits, with the first based on overstory density and the second based on soil moisture. Class 4 was characterized by frequent fire, forested history, high basal area (≥9.965 m^2^/ha), and lower soil moisture (<42.12%), class 5 by frequent fire, forested history, high basal area, and higher soil moisture (≥42.12%), and class 6 by frequent fire, forested history, and low basal area (<9.965 m^2^/ha).

### Location-specific Classifications

Location-specific classifications illustrated a number of differences from the All Sites classification, in terms of the identity and ordering of degrading factors, as well as the role of soils (Figure 3). The Fort Bragg classification contained a single split and two groups defined by agricultural land-use history. The Fort Stewart classification contained three splits and four groups, which illustrated a prominent influence of soils. The first split was between Entisol/Ultisol sites and Inceptisol/Spodosol sites. Inceptisol/Spodosol sites (Class 1) formed a unique soil-vegetation association in the model, which was not related to degradation. The only degrading factor in this model, non-*Pinus* basal area, distinguished among sites on Entisol and Ultisol soils (low basal area sites were further split by soil moisture). The Savannah River Site classification contained splits based on all three degrading factors, but with differing order of importance relative to the All Sites classification, as basal area was the most important factor, followed by agricultural land-use history.

### Degradation Classes in Relation to Reference Conditions

**Figure 4:**
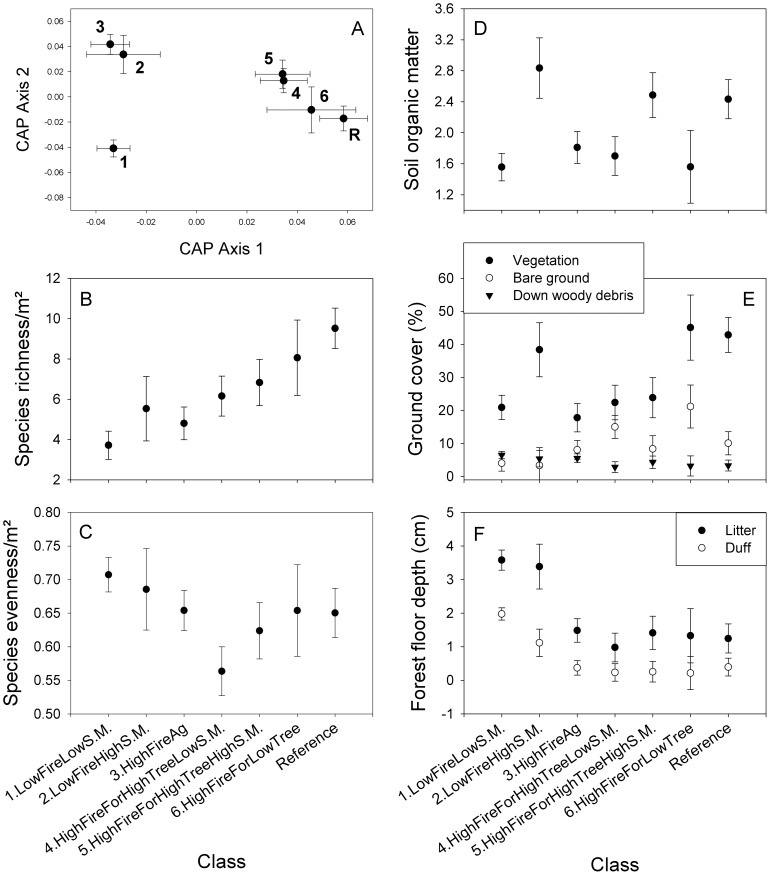
Comparison of Classes (1–6) from the All Sites classification and regression tree analyses to reference conditions. A) Understory community composition, B) understory species richness, C) understory species evenness, D) soil organic matter content, E) ground cover variables, and F) forest floor depth. All values are means ±95% confidence intervals.

The All Sites classification revealed three main delineations in community composition among classes: sites with forested history (Classes 4–6, reference sites), sites with agricultural history or sites with infrequent fire and high soil moisture (Classes 2, 3), and sites with infrequent fire and low soil moisture (Class 1) (Figure 4a). Species richness declined with degradation, whereas species evenness was generally greater at infrequently burned sites (Figure 4b,c). Among ground cover attributes, vegetation cover was greatest at sites with high soil moisture and at reference sites (Figure 4e), bare ground was greatest at two of the frequently burned classes with forested history, and little difference in down woody debris was apparent (Figure 4e). Forest floor accumulation was related to fire suppression, with greater depths of litter and duff in Classes 1 and 2 (Figure 4f). Soil organic matter was markedly higher on sites with high soil moisture and at reference sites, but not strongly associated with degradation (Figure 4d; Table 2).

**Table 2:** Attributes of degradation classes (1–6) for the All Sites classification and reference sites.

Class	Canopy cover (%)	Total basal area(m^2^/ha)	Pinus basal area(m^2^/ha)	Non-Pinus basalarea (m^2^/ha)	Years since fire	# Fires1991–2009	Soil water holding capacity (%)
1. Low fire/Low SM^1^	62.4±4.2	20.3±1.6	18.2±1.6	2.0±0.7	7.6±1.2	2.3±0.4	37.0±1.3
2. Low fire/High SM	63.0±9.4	18.3±3.5	16.2±3.5	2.2±1.7	3.1±2.6	2.7±0.8	48.9±3.0
3. Ag/High fire	51.8±4.9	20.5±1.8	18.5±1.9	2.0±0.9	1.0±1.3	5.9±0.4	38.9±1.55
4. Forest/High Fire/High BA^2^/Low SM	34.8±6.0	16.2±2.2	14.8±2.6	1.4±1.1	0.8±1.6	6.4±0.5	37.2±1.9
5. Forest/High Fire/High BA/High SM	45.7±7.0	19.6±2.6	18.2±2.6	1.3±1.2	1.0±1.9	6.4±0.6	46.3±2.2
6. Forest/High fire/Low BA	35.1±11.3	7.3±4.2	7.0±4.3	0.4±2.0	1.5±3.1	5.7±1.0	37.9±3.6
Reference	44.4±6.1	15.3±2.4	14.0±2.3	1.3±1.1	1.0±1.7	6.0±0.5	46.6±2.3

Values are mean ±95% confidence interval.

1 Soil moisture holding capacity.

2 Basal area.

Models resulting from the location-specific classifications produced classes that were compositionally distinct, with the exception of Classes 1 and 3 at Fort Stewart (Figures S1, S2, and S3). Reference sites ranged from compositionally unique at Fort Bragg (Figure S1) to comparable to less degraded classes on at least one CAP axis at Fort Stewart and Savannah River Site (Figures S2, S3). Species richness declined with degradation in each location-specific model, whereas many other attributes illustrated location-specific patterns (Figures S1, S2, and S3, Table S5). Metrics used to classify sites (e.g., years since last fire, basal area; Table S5) largely mirrored each classification; however, the ways that these variables changed with degradation varied with the different classification rules at each location. Several variables were associated with degradation at one or more sites, including soil organic matter, vegetation cover, and forest floor depth at Savannah River Site, vegetation cover at Fort Bragg, and forest floor depth at Fort Stewart. Other metrics, such as species evenness and soil variables, illustrated location-specific patterns.

## Discussion

### An Ecological Reference Model for Longleaf Pine Woodlands

Ecological reference models are commonly employed during restoration, but are frequently qualitative, which limits their utility for prioritizing sites for restoration and guiding restoration efforts on the ground [4]. Using data from 232 sites, we quantified an ecological reference model for longleaf pine woodland understory plant communities spanning a broad geographic region of the Southeastern United States (Figure 3a). Three degrading factors – agricultural legacies, recent fire history, and overstory tree abundance – as well as soil moisture holding capacity, delineated classes of plant communities in this regional model. Location-specific models, however, illustrated substantial variation relative to this regional model and each other (Figure 3). We suggest that our regional model can help prioritize longleaf pine woodlands for restoration across our study region; however, due to substantial landscape-to-landscape variation, location-specific reference models can help guide local management decisions.

Our regional model provides general support for the core components of a qualitative model of longleaf pine degradation (Figure 1); however, by quantifying this conceptual model, we illustrate the relative contributions of degrading factors, as well as threshold values that determine differences between degraded classes. In this regional model, fire frequency was the most important factor (i.e., the first split in the classification), followed by agricultural legacies (Figure 3a). Overstory basal area, while a significant factor in the regional model, was the least important. Based on similarity in community composition to reference sites, Classes 1–3 (infrequently burned and post-agricultural sites) were the most degraded, whereas historically forested sites were less degraded, being both compositionally similar to each other and to reference sites (Figure 4a). Our regional model further quantifies 4 vs. 5 fires since 1991 and ∼10 m^2^/ha as breakpoints between degraded classes related to burning and tree basal area, respectively. These results reinforce the importance of frequent prescribed fires for management of longleaf pine woodlands [8], [13], because of its positive influence on understory plant communities [15], [17]. Our results further highlight the role of agricultural legacies in the degradation of longleaf pine plant communities [14]–[16]. The pronounced deviation of fire suppressed and post-agricultural plant communities from reference conditions (Figure 4) suggests that sites supporting these degraded conditions may require the greatest efforts to restore.

The resulting classes from our regional and location-specific analyses captured relevant gradients of degradation (Figures 4, S1, S2, and S3). As degradation increased, levels of biotic and abiotic variables became more dissimilar from values at reference sites. This pattern was consistent across the regional and location-specific models for several variables including understory richness, cover, and composition, forest floor accumulation, and tree basal area, whereas other variables including soil organic matter showed a degradation gradient in one or more models. Importantly, a number of these variables were not used in the construction of our ecological reference models (e.g., species diversity metrics, ground cover components, forest floor depth, and soil attributes), yet in many cases these variables corresponded with the degradation gradient.

The explicit consideration of land-use legacies has received increasing attention in conservation and restoration [3], [10], [24], [44], [45]. Our results illustrate how multiple drivers of ecosystem degradation, including land-use legacies, can be quantitatively incorporated into ecological reference models. This may be of particular importance when legacies interact with contemporary management to influence population viability and community composition. For example, past agricultural activities can mediate the effects of present-day fire management on plant community diversity, such that frequent burning may lead to increased richness, but only in sites with an agricultural land-use history [15]. Similarly, past land use can modify levels and patterns of soil nutrients, with ensuing effects on plant populations [46]. Future research will be necessary before specific restoration strategies might be tailored to the various degraded states in our regional and location-specific models (see below) and our work shows that consideration of both contemporary and historical causes of degradation will be important during this process.

### Generality of the Ecological Reference Model

Our location-specific models illustrated notable variation, both relative to each other and to the regional All Sites model (Figure 3). Only one location-specific model (Savannah River Site) contained all three degrading factors, but with the reverse order of importance compared to the regional model. Conversely, Fort Bragg supported a simple two class model based only on agricultural land-use history, whereas Fort Stewart’s model illustrated a prominent role of soils, in addition to hardwood abundance, for structuring understory plant communities (Figure 3). This variation may reflect differences in underlying environmental factors or differences in land-use and management histories among our three study locations (Table 1). For example, the presence or absence of fire frequency as a variable in location-specific models may be explained in part by variation in prescribed fire management among locations (Table 1). At Fort Bragg, where fire frequency was not selected as a model component, prescribed fire is highly regimented, resulting in frequent fires and little variation in time since fire among sites (Figure 3). Conversely, substantial variation in fire frequency exists among sites at the Savannah River Site (Table 1), where fire frequency was selected as a model component (Figure 3). Furthermore, our study spanned three physiographic regions of the southeastern U.S. coastal plain [26], capturing variation in important ecological factors such as dominant species (Tables S2, S3, and S4) and soil conditions (Table 1) [26], [33]. Longleaf pine communities are broadly structured by soils [26] and this influence of soils was most prominent at Fort Stewart – a location that supports longleaf pine woodlands underlain by a variety of soil orders, including Spodosols and Inceptisols, which formed a unique class in the Fort Stewart model. Fort Stewart also illustrated the lone example of how the influence of a degrading factor depended on soil conditions, as non-*Pinus* basal area was important to plant communities on Entisol and Ultisol soils, but not Inceptisol and Spodosol soils. Importantly, at least one hypothesized degrading factors (Figure 1) was a significant model component at each location, illustrating that these factors structured understory degradation (i.e., departure from reference conditions) at both regional and landscape scales in this study. The differences in reference model details between our three study locations (Figure 3) suggests that, while our regional reference model may provide broad-scale insight into patterns of longleaf pine degradation, locations with available resources should consider collecting data to parameterize location-specific reference models.

### Implications for Conservation and Restoration

We suggest that our regional ecological reference model provides a way for managers to broadly infer the degradation status of longleaf pine understory communities in our study region. This model, as well as the location-specific models, employs relatively easily measured data, which serve as proxies for ecological characteristics of interest to land managers (Figure 4, S1, S2, and S3). Agricultural and fire history and, perhaps, overstory basal area, may be available to land managers as GIS data. Thus, our regional and location-specific reference models may be mapped at large spatial scales to assist management and conservation decisions. Longleaf pine understory communities are notable for high levels of species diversity from local (e.g., 1 m×1 m) to regional scales [13], [26]. Our findings provide guidance over much of this range in scales, spanning sites, landscapes, and portions of a region (the Atlantic coastal plain). Future work might explore additional reference models to inform small-scale (e.g., within site) restoration decisions.

Our approach to classifying communities based on degraded conditions should be broadly applicable to other ecosystems around the world modified by human land use and altered fire regimes, including fragmented woodlands in Australia [45], fire-suppressed savannas in Brazil [47], and forests in the western United States [6], [48], among others. During such application, our framework for developing reference models should be modified to include the relevant suspected drivers of degradation for an ecosystem of interest. For example, the presence of invasive species is an important consideration during longleaf pine woodland restoration [8] and, while invasive species were not abundant at our study sites, their inclusion as a model variable might be important at other longleaf pine sites or in other ecosystems.

The remaining challenge is to determine how to best restore longleaf pine understory communities once patterns of degradation have been assessed. Our regional and location-specific ecological reference models suggest some strategies for restoration, but these should be interpreted with caution. For example, transitioning sites between Classes 4/5 and 6 in our regional model might simply entail mechanical thinning of overstory trees, a strategy that can increase plant diversity in some contexts [49] but has limited effects in others [20], [50]. Other transitions might require multiple restoration strategies. For example, restoration of fire regime at post-agricultural sites (e.g., transitioning between Classes 2 and 3 at Savannah River Site) might need to be coupled with introduction of propagules – particularly those of dispersal-limited plant groups, such as passive and ant dispersed species like *Tephrosia virginiana* and *Aristida stricta*/*beyrichiana* [51] (Tables S1, S2, S3, and S4). Future experimental work will be necessary to evaluate these hypotheses suggested by our models. Further, reinstating processes may lead to unexpected outcomes for some degraded states. For example, reintroducing fire to long-unburned sites may produce novel fire behavior, leading to unexpected outcomes, such as mortality of longleaf pine overstory trees [52]. Moreover, while our conceptual reference model (Figure 1) depicts a simplistic set of linear transitions among degraded states, it remains an open question as to whether a simple linear or a non-linear, such as alternative stable states [53] or state and transition (e.g. [54]), model of restoration will be most appropriate during restoration of longleaf pine woodlands. Finally, more work is needed to understand how to best tailor combinations and sequences of restoration strategies (e.g., seed addition, prescribed fire, overstory thinning) to the variety of degraded conditions illustrated by our reference models for longleaf pine woodlands.

## Supporting Information

Figure S1 **Comparison of Classes (1–2) from the Fort Bragg classification and regression tree analyses to reference conditions.** A) understory community composition, B) understory species richness, C) understory species evenness, D) soil organic matter content, E) ground cover variables, and F) forest floor depth. All values are means ±95% confidence intervals.
(TIF)

Figure S2 **Comparison of Classes (1–4) from the Fort Stewart classification and regression tree analyses to reference conditions.** A) understory community composition, B) understory species richness, C) understory species evenness, D) soil organic matter content, E) ground cover variables, and F) forest floor depth. All values are means ±95% confidence intervals.
(TIF)

Figure S3 **Comparison of Classes (1–4) from the Savannah River Site classification and regression tree analyses to reference conditions.** A) understory community composition, B) understory species richness, C) understory species evenness, D) soil organic matter content, E) ground cover variables, and F) forest floor depth. All values are means ±95% confidence intervals.
(TIF)

Table S1 **Species with the 10 highest indicator values (from Indicator Species Analysis) for each site class in the All Sites classification.** Species identified as indicators of individual site classes (p<0.05) are noted by *. P-values are listed for significant indicator species; where not provided p-values were ≥0.05. The number of significant indicator species decreased strongly with degradation; Classes 1–6 contained 2, 18, 0, 3, 0, 40 significant indicator species, respectively. Thirteen species were indicative of reference sites.
(DOCX)

Table S2 **Species with the 10 highest indicator values (from Indicator Species Analysis) for each site class in the Fort Bragg classification.** Species identified as indicators of individual site classes are noted by *. Indicator species were primarily present at reference sites (n = 59), with 0 and 4 in Classes 1 and 2, respectively.
(DOCX)

Table S3 **Species with the 10 highest indicator values (from Indicator Species Analysis) for each site class in the Fort Stewart classification.** Species identified as indicators of individual site classes are noted by *. 7, 1, 9, and 10 species were significant indicators of classes 1–4, respectively, and 19 species were indicative of reference sites.
(DOCX)

Table S4 **Species with the 10 highest indicator values (from Indicator Species Analysis) for each site class in the Savannah River Site classification.** Species identified as indicators of individual site classes are noted by *. There were 0, 3, 14, and 4 significant indicators of classes 1–4, respectively, and 26 species indicative of reference sites.
(DOCX)

Table S5 **Attributes of classes resulting from the location-specific classifications, compared to references sites.** Values are mean ±95% confidence interval.
(DOCX)
