# Supplementary figures and images for: Land-Use History and Contemporary Management Inform an Ecological Reference Model for Longleaf Pine Woodland Understory Plant Communities
Source: PLoS One. 2014 Jan 23;9(1):e86604. doi: 10.1371/journal.pone.0086604 (PMC3900602; doi:10.1371/journal.pone.0086604)

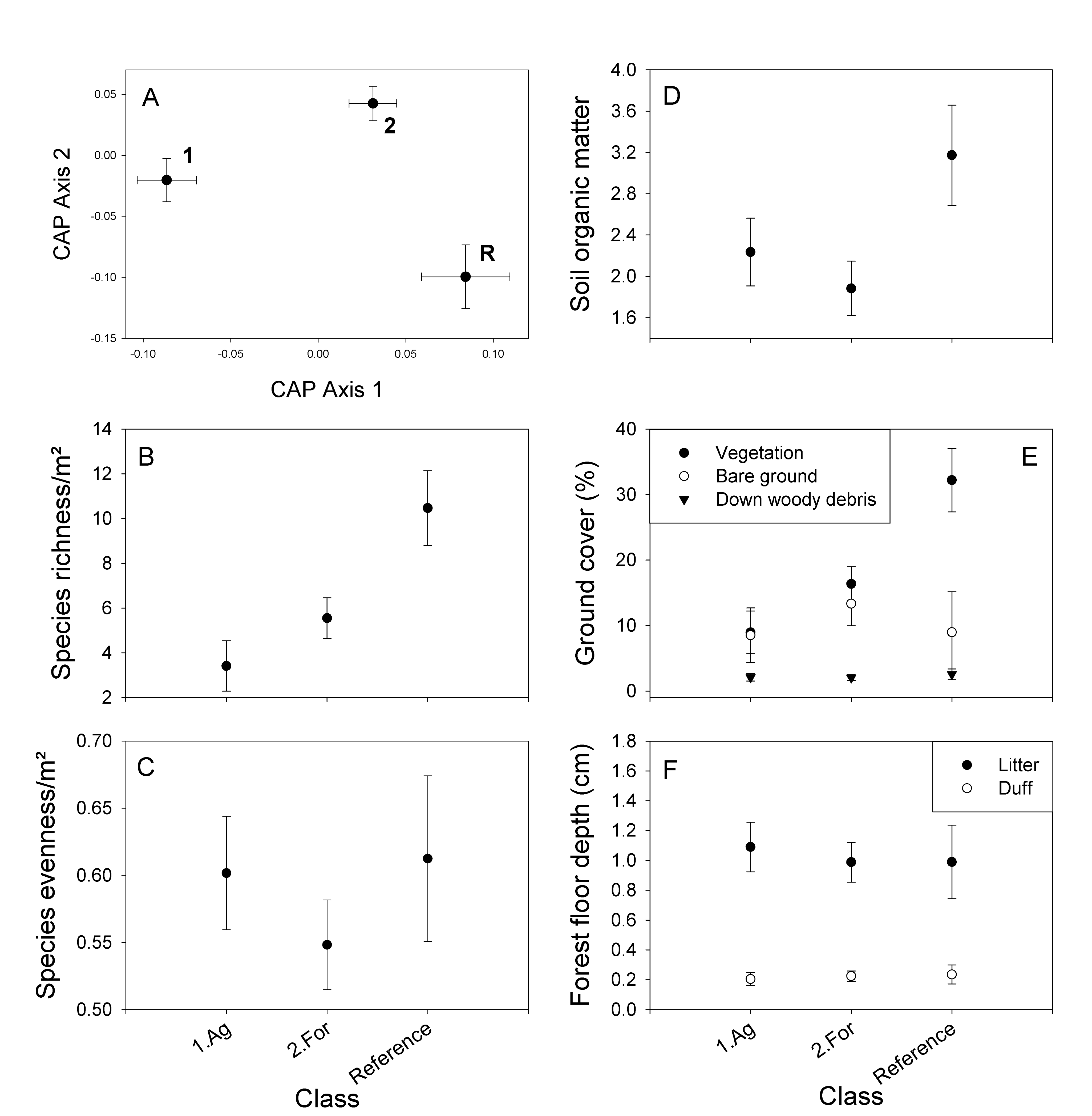

Supplement: Figure S1 — Comparison of Classes (1–2) from the Fort Bragg classification and regression tree analyses to reference conditions. A) understory community composition, B) understory species richness, C) understory species evenness, D) soil organic matter content, E) ground cover variables, and F) forest floor depth. All values are means ±95% confidence intervals. (TIF) [file pone.0086604.s001.tif]

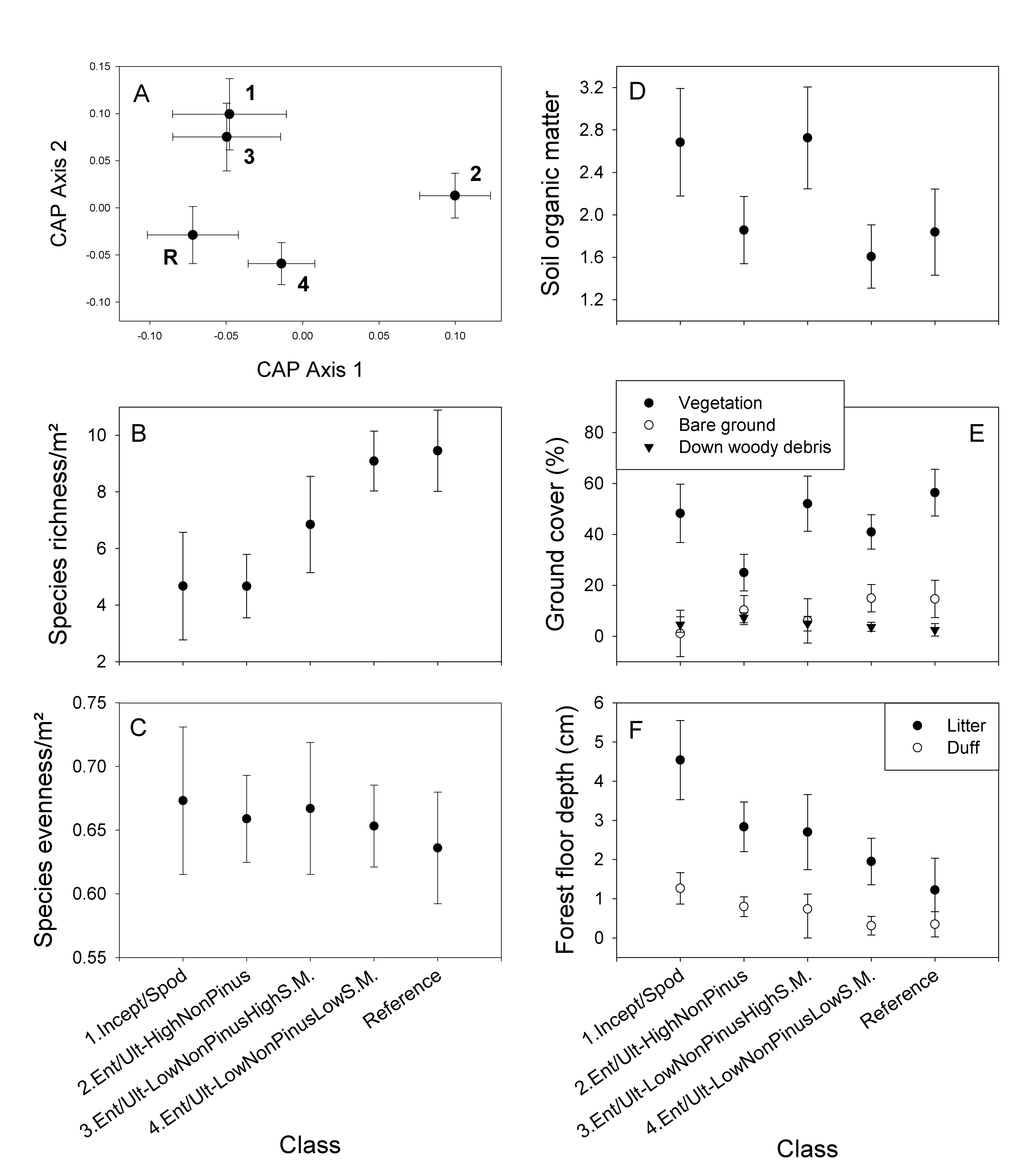

Supplement: Figure S2 — Comparison of Classes (1–4) from the Fort Stewart classification and regression tree analyses to reference conditions. A) understory community composition, B) understory species richness, C) understory species evenness, D) soil organic matter content, E) ground cover variables, and F) forest floor depth. All values are means ±95% confidence intervals. (TIF) [file pone.0086604.s002.tif]

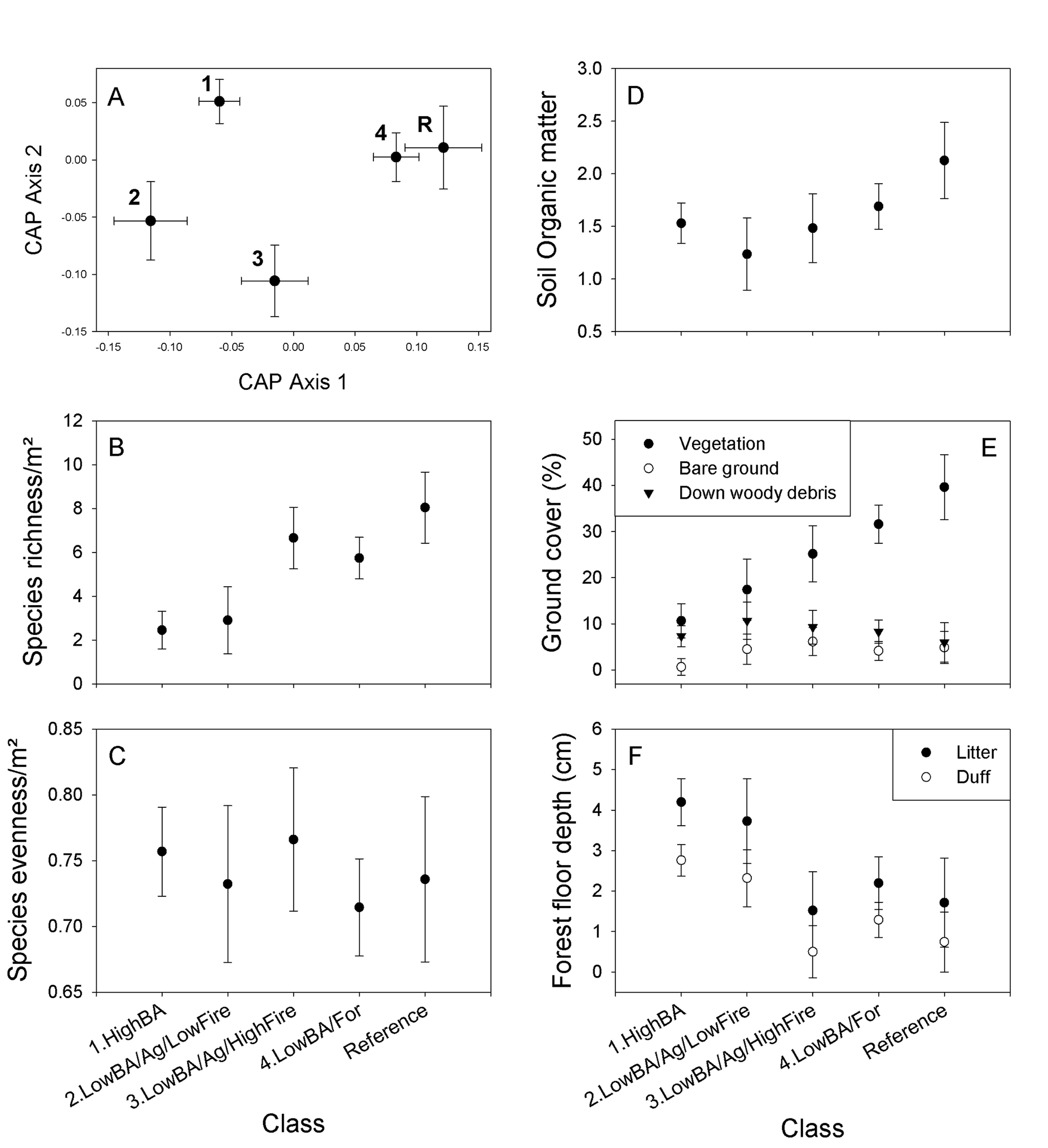

Supplement: Figure S3 — Comparison of Classes (1–4) from the Savannah River Site classification and regression tree analyses to reference conditions. A) understory community composition, B) understory species richness, C) understory species evenness, D) soil organic matter content, E) ground cover variables, and F) forest floor depth. All values are means ±95% confidence intervals. (TIF) [file pone.0086604.s003.tif]
