# Supplementary material for: Land-Use History and Contemporary Management Inform an Ecological Reference Model for Longleaf Pine Woodland Understory Plant Communities
Source: PLoS One. 2014 Jan 23;9(1):e86604. doi: 10.1371/journal.pone.0086604 (PMC3900602; doi:10.1371/journal.pone.0086604)
Supplement: Table S1 — Species with the 10 highest indicator values (from Indicator Species Analysis) for each site class in the All Sites classification. Species identified as indicators of individual site classes (p<0.05) are noted by *. P-values are listed for significant indicator species; where not provided p-values were ≥0.05. The number of significant indicator species decreased strongly with degradation; Classes 1–6 contained 2, 18, 0, 3, 0, 40 significant indicator species, respectively. Thirteen species were indicative of reference sites. (DOCX) [file pone.0086604.s004.docx]

| Class | Species | Indicator value | P |
| --- | --- | --- | --- |
| 1. Low fire/ Low SM^1^ | *Vitis rotundifolia** | 23.5 | 0.006 |
|  | *Vaccinium stamineum** | 17.0 | 0.04 |
|  | *Gelsemium sempervirens* | 13.0 |  |
|  | *Tragia urens* | 9.5 |  |
|  | *Smilax glauca* | 9.3 |  |
|  | *Sassafras albidum* | 9.0 |  |
|  | *Rhus toxicodendron* | 7.0 |  |
|  | *Quercus laurifolia* | 7.2 |  |
|  | *Crataegus flava* | 7.0 |  |
|  | *Vaccinium arboreum* | 6.0 |  |
| 2. Low fire/ High SM | *Vaccinium atrococcum** | 36.3 | 0.0002 |
|  | *Quercus nigra** | 26.6 | 0.001 |
|  | *Rhexia mariana** | 26.2 | 0.002 |
|  | *Gaylussacia frondosa** | 21.0 | 0.003 |
|  | *Ilex glabra** | 20.9 | 0.004 |
|  | *Lyonia mariana** | 20.4 | 0.003 |
|  | *Pinus* species (excluding *P. palustris*) * | 19.2 | 0.01 |
|  | *Lyonia lucida** | 15.5 | 0.005 |
|  | *Oldenlandia uniflora** | 15.2 | 0.004 |
|  | *Panicum virgatum** | 15.0 | 0.007 |
| 3. Ag/High fire | *Vitis rotundifolia* | 15.0 |  |
|  | *Dichanthelium* species | 14.0 |  |
|  | *Diospyros virginiana* | 10.6 |  |
|  | *Andropogon* species | 10.0 |  |
|  | *Sassafras albidum* | 9.7 |  |
|  | *Pinus palustris* | 9.0 |  |
|  | *Paspalum setaceum* | 9.1 |  |
|  | *Rubus flagellaris* | 7.8 |  |
|  | *Eupatorium compositifolium* | 7.0 |  |
|  | *Prunus serotina* | 7.3 |  |
| 4. Forest/High Fire/High BA^2^/Low SM | *Quercus laevis** | 40.6 | 0.0002 |
|  | *Cnidoscolus stimulosus** | 23.5 | 0.005 |
|  | *Gaylussacia dumosa* | 18.0 |  |
|  | *Liatris regimontis** | 17.5 | 0.009 |
|  | *Rhus toxicodendron* | 12.3 |  |
|  | *Tephrosia virginiana* | 12.0 |  |
|  | *Aristida stricta* | 11.0 |  |
|  | *Andropogon* species | 9.0 |  |
|  | *Silphium compositum* | 7.6 |  |
|  | *Heterotheca graminifolia* | 7.0 |  |
| 5. Forest/High Fire/High BA/High SM | *Aristida stricta* | 18.0 |  |
|  | *Gaylussacia dumosa* | 14.0 |  |
|  | *Diospyros virginiana* | 10.0 |  |
|  | *Andropogon* species | 9.0 |  |
|  | *Euphorbia curtisii* | 8.8 |  |
|  | *Quercus laevis* | 8.0 |  |
|  | *Quercus margaretta* | 8.5 |  |
|  | *Solidago odora* | 7.0 |  |
|  | *Eupatorium hyssopifolium* | 6.7 |  |
|  | *Vaccinium myrsinites* | 7.0 |  |
| 6. Forest/High fire/Low BA | *Rhus copallina** | 34.2 | 0.0006 |
|  | *Diodia teres** | 30.5 | 0.0004 |
|  | *Heterotheca graminifolia** | 29.1 | 0.002 |
|  | *Hypericum gentianoides** | 28.4 | 0.0004 |
|  | *Lechea villosa** | 27.6 | 0.0002 |
|  | *Dichanthelium* species* | 26.7 | 0.003 |
|  | *Bonamia patens** | 23.8 | 0.002 |
|  | *Aristida stricta** | 21.3 | 0.02 |
|  | *Cassia fasciculata** | 21.1 | 0.004 |
|  | *Andropogon* species* | 20.0 |  |
| Reference | *Andropogon* species* | 24.1 | 0.03 |
|  | *Aristida stricta* | 21.0 |  |
|  | *Gaylussacia dumosa* | 21.3 |  |
|  | *Stylosanthes biflora** | 20.5 | 0.01 |
|  | *Aristida purpurascens** | 19.5 | 0.01 |
|  | *Aster squarrosus** | 16.0 | 0.01 |
|  | *Vaccinium crassifolium** | 15.8 | 0.002 |
|  | *Aster linariifolius** | 15.7 | 0.01 |
|  | *Heterotheca graminifolia* | 14.0 |  |
|  | *Tephrosia virginiana* | 13.9 |  |

^1^ Soil moisture holding capacity.

^2^ Basal area
