# Supplementary material for: Land-Use History and Contemporary Management Inform an Ecological Reference Model for Longleaf Pine Woodland Understory Plant Communities
Source: PLoS One. 2014 Jan 23;9(1):e86604. doi: 10.1371/journal.pone.0086604 (PMC3900602; doi:10.1371/journal.pone.0086604)
Supplement: Table S2 — Species with the 10 highest indicator values (from Indicator Species Analysis) for each site class in the Fort Bragg classification. Species identified as indicators of individual site classes are noted by *. Indicator species were primarily present at reference sites (n = 59), with 0 and 4 in Classes 1 and 2, respectively. (DOCX) [file pone.0086604.s005.docx]

| Class | Species | Indicator value | P |
| --- | --- | --- | --- |
| 1. Ag | *Pinus palustris* | 26.3 |  |
|  | *Dichanthelium* species | 18.0 |  |
|  | *Danthonia sericea* | 15.9 |  |
|  | *Vaccinium tenellum* | 15.0 |  |
|  | *Diospyros virginiana* | 15.0 |  |
|  | *Sassafras albidum* | 13.7 |  |
|  | *Quercus laevis* | 13.0 |  |
|  | *Quercus marilandica* | 12.0 |  |
|  | *Andropogon* species | 12.0 |  |
|  | *Pinus* species (excluding *P. palustris*) | 9.5 |  |
| 2. Forest | *Quercus laevis** | 48.8 | 0.002 |
|  | *Gaylussacia dumosa** | 47.3 | 0.007 |
|  | *Rhus toxicodendron** | 31.9 | 0.04 |
|  | *Aristida stricta* | 30.0 |  |
|  | *Pinus palustris* | 23.0 |  |
|  | *Tragia urens* | 19.7 |  |
|  | *Andropogon* species | 19.0 |  |
|  | *Cnidoscolus stimulosus* | 18.3 |  |
|  | *Euphorbia ipecacuanhae* | 18.2 |  |
|  | *Tephrosia virginiana* | 18.0 |  |
| Reference | *Andropogon* species* | 61.8 | 0.0002 |
|  | *Aristida stricta** | 56.4 | 0.0002 |
|  | *Cassia nictitans** | 44.6 | 0.0002 |
|  | *Eupatorium album** | 40.6 | 0.0002 |
|  | *Erigeron canadensis** | 40.0 | 0.0002 |
|  | *Vaccinium tenellum** | 39.9 | 0.006 |
|  | *Vaccinium crassifolium** | 39.9 | 0.0002 |
|  | *Dichanthelium* species* | 38.9 | 0.01 |
|  | *Scleria ciliata** | 38.9 | 0.002 |
|  | *Aster squarrosus** | 38.1 | 0.0002 |
