# Supplementary material for: Land-Use History and Contemporary Management Inform an Ecological Reference Model for Longleaf Pine Woodland Understory Plant Communities
Source: PLoS One. 2014 Jan 23;9(1):e86604. doi: 10.1371/journal.pone.0086604 (PMC3900602; doi:10.1371/journal.pone.0086604)
Supplement: Table S3 — Species with the 10 highest indicator values (from Indicator Species Analysis) for each site class in the Fort Stewart classification. Species identified as indicators of individual site classes are noted by *. 7, 1, 9, and 10 species were significant indicators of classes 1–4, respectively, and 19 species were indicative of reference sites. (DOCX) [file pone.0086604.s006.docx]

| Class | Species | Indicator value | P |
| --- | --- | --- | --- |
| 1. Inceptisols, Spodosols | *Gaylussacia frondosa** | 52.0 | 0.0002 |
|  | *Persea palustris** | 42.2 | 0.003 |
|  | *Serenoa repens** | 27.4 | 0.03 |
|  | *Lyonia ferruginea/fruticosa** | 25.5 | 0.02 |
|  | *Lyonia lucida** | 24.8 | 0.01 |
|  | *Clethra alnifolia** | 24.7 | 0.01 |
|  | *Cyperus globulosus** | 19.2 | 0.02 |
|  | *Ilex glabra** | 17.0 |  |
|  | *Vaccinium myrsinites** | 15.0 |  |
|  | *Gaylussacia dumosa** | 14.0 |  |
| 2. Entisols, Ultisols/ High non-*pinus* Basal area | *Vitis rotundifolia** | 32.3 | 0.02 |
|  | *Gelsemium sempervirens* | 20.0 |  |
|  | *Cyperus retrorsus* | 18.5 |  |
|  | *Smilax rotundifolia* | 15.8 |  |
|  | *Uniola laxa* | 14.4 |  |
|  | *Callicarpa americana* | 13.3 |  |
|  | *Carex retroflexa* | 13.0 |  |
|  | *Cassia fasiculata* | 12.0 |  |
|  | *Rubus trivialis* | 11.0 |  |
|  | *Pinus* species (excluding *P. palustris*) | 11.0 |  |
| 3. Entisols, Ultisols/ Low non-*pinus* Basal area/ High soil moisture | *Vaccinium atrococcum** | 62.6 | 0.0002 |
|  | *Ilex glabra** | 38.3 | 0.002 |
|  | *Rhexia mariana** | 37.1 | 0.01 |
|  | *Rhexia alifanus** | 33.8 | 0.004 |
|  | *Vaccinium myrsinites* | 33.6 |  |
|  | *Liquidambar styraciflua** | 31.6 | 0.005 |
|  | *Pinus* species (excluding *P. palustris*) * | 29.9 | 0.01 |
|  | *Aster paternus** | 27.9 | 0.003 |
|  | *Lachnocaulon anceps** | 26.5 | 0.01 |
|  | *Dichanthelium* species | 24.0 |  |
| 4. Entisols, Ultisols/ Low non-*pinus* Basal area / Low soil moisture | *Scleria ciliata** | 39.2 | 0.004 |
|  | *Heterotheca graminifolia** | 37.5 | 0.007 |
|  | *Diodia teres** | 36.2 | 0.004 |
|  | *Pinus palustris** | 34.2 | 0.007 |
|  | *Eupatorium compositifolium** | 31.4 | 0.03 |
|  | *Rubus trivialis** | 30.3 | 0.02 |
|  | *Andropogon* species | 27.0 |  |
|  | *Dichanthelium* species | 25.0 |  |
|  | *Aristida stricta* | 25.0 |  |
|  | *Eupatorium album** | 23.9 | 0.03 |
| Reference | *Aristida stricta** | 46.8 | 0.0008 |
|  | *Quercus pumila** | 34.3 | 0.007 |
|  | *Aristida purpurascens** | 32.6 | 0.01 |
|  | *Heterotheca graminifolia* | 32.0 |  |
|  | *Andropogon* species | 31.3 |  |
|  | *Gaylussacia dumosa* | 27.4 |  |
|  | *Aster squarrosus** | 27.3 | 0.009 |
|  | *Seymeria cassioides** | 26.5 | 0.01 |
|  | *Hypericum hypericoides** | 24.9 | 0.03 |
|  | *Xyris caroliniana** | 24.8 | 0.008 |
