# Supplementary material for: Land-Use History and Contemporary Management Inform an Ecological Reference Model for Longleaf Pine Woodland Understory Plant Communities
Source: PLoS One. 2014 Jan 23;9(1):e86604. doi: 10.1371/journal.pone.0086604 (PMC3900602; doi:10.1371/journal.pone.0086604)
Supplement: Table S4 — Species with the 10 highest indicator values (from Indicator Species Analysis) for each site class in the Savannah River Site classification. Species identified as indicators of individual site classes are noted by *. There were 0, 3, 14, and 4 significant indicators of classes 1–4, respectively, and 26 species indicative of reference sites. (DOCX) [file pone.0086604.s007.docx]

| Class | Species | Indicator value | P |
| --- | --- | --- | --- |
| 1. High basal area | *Vitis rotundifolia* | 17.0 |  |
|  | *Sassafras albidum* | 12.0 |  |
|  | *Crataegus flava* | 11.5 |  |
|  | *Vaccinium stamineum* | 10.0 |  |
|  | *Chimaphila maculata* | 9.4 |  |
|  | *Lespedeza stuevei* | 9.4 |  |
|  | *Quercus laurifolia* | 7.0 |  |
|  | *Diospyros virginiana* | 6.0 |  |
|  | *Quercus falcata* | 6.0 |  |
|  | *Carya tomentosa* | 6.0 |  |
| 2. Ag/Low fire/Low basal area | *Vitis rotundifolia** | 35.2 | 0.008 |
|  | *Gelsemium sempervirens** | 32.0 | 0.02 |
|  | *Quercus incana** | 23.0 | 0.05 |
|  | *Tragia urens* | 21.3 |  |
|  | *Smilax glauca* | 19.7 |  |
|  | *Vaccinium stamineum* | 15.0 |  |
|  | *Rhus toxicodendron* | 10.0 |  |
|  | *Lupinus diffusus* | 10.0 |  |
|  | *Aristida tuberculosa* | 10.0 |  |
|  | *Rubus flagellaris* | 10.0 |  |
| 3. Ag/High fire/Low basal area | *Cassia nictitans** | 70.7 | 0.0002 |
|  | *Desmodium marilandicum** | 51.1 | 0.0002 |
|  | *Prunus serotina** | 37.5 | 0.008 |
|  | *Dichanthelium* species*** | 35.1 | 0.03 |
|  | *Rubus flagellaris** | 33.3 | 0.007 |
|  | *Pinus palustris** | 31.7 | 0.02 |
|  | *Erianthus brevibarbis** | 29.1 | 0.002 |
|  | *Rhus toxicodendron** | 28.8 | 0.05 |
|  | *Sassafras albidum* | 27.0 |  |
|  | *Rhus copallina* | 24.9 |  |
| 4. Forest/Low basal area | *Gaylussacia dumosa* | 36.0 |  |
|  | *Quercus laevis** | 23.9 | 0.04 |
|  | *Pteridium aquilinum** | 23.7 | 0.03 |
|  | *Rhus copallina* | 21.0 |  |
|  | *Vaccinium arboreum* | 21.0 |  |
|  | *Vernonia angustifolia** | 19.3 | 0.04 |
|  | *Baptisia perfoliata** | 19.2 | 0.03 |
|  | *Vaccinium stamineum* | 19.1 |  |
|  | *Quercus stellata* | 19.0 |  |
|  | *Andropogon* species | 18.0 |  |
|  | *Dichanthelium* species | 18.0 |  |
| Reference | *Gaylussacia dumosa** | 48.0 | 0.001 |
|  | *Tephrosia virginiana** | 45.2 | 0.0004 |
|  | *Andropogon* species*** | 37.6 | 0.02 |
|  | *Lespedeza repens** | 37.1 | 0.002 |
|  | *Dyschoriste oblongifolia** | 35.9 | 0.002 |
|  | *Eupatorium album** | 34.9 | 0.002 |
|  | *Solidago odora** | 32.3 | 0.02 |
|  | *Aster linariifolius** | 31.6 | 0.003 |
|  | *Stipa avenacea** | 31.6 | 0.002 |
|  | *Aster paternus** | 29.4 | 0.003 |
