# Supplementary material for: Land-Use History and Contemporary Management Inform an Ecological Reference Model for Longleaf Pine Woodland Understory Plant Communities
Source: PLoS One. 2014 Jan 23;9(1):e86604. doi: 10.1371/journal.pone.0086604 (PMC3900602; doi:10.1371/journal.pone.0086604)
Supplement: Table S5 — Attributes of classes resulting from the location-specific classifications, compared to references sites. Values are mean ±95% confidence interval. (DOCX) [file pone.0086604.s008.docx]

|  | Class | Canopy cover (%) | Total basal area (m²/ha) | Pinus basal area (m²/ha) | Non-Pinus basal area (m²/ha) | Years since fire | # Fires since 1991 | Soil water holding capacity (%) |
| --- | --- | --- | --- | --- | --- | --- | --- | --- |
| Fort Bragg |  |  |  |  |  |  |  |  |
|  | 1. Ag | 33.9±2.9 | 21.9±2.3 | 19.3±2.7 | 2.6±1.2 | 1.0±0.4 | 5.5±0.5 | 42.4±2.5 |
|  | 2. Forest | 28.8±2.4 | 17.8±1.8 | 16.5±2.2 | 1.3±1.0 | 1.0±0.3 | 6.0±0.4 | 40.3±2.0 |
|  | Reference | 34.0±4.1 | 18.8±2.5 | 17.7±3.4 | 1.1±1.8 | 1.0±0.4 | 4.9±0.6 | 55.0±3.7 |
| Fort Stewart |  |  |  |  |  |  |  |  |
|  | 1. Inceptisols, Spodosols | 66.0±12.8 | 15.9±4.9 | 14.6±4.7 | 1.4±1.3 | 4.0±1.8 | 4.1±1.7 | 43.5±3.6 |
|  | 2. Entisols, Ultisols/ High non-Pinus BA^1^ | 71.3±8.0 | 18.9±3.1 | 14.8±2.9 | 4.1±0.6 | 3.1±1.0 | 5.1±1.1 | 38.5±2.2 |
|  | 3. Entisols, Ultisols/ Low non-Pinus BA/ High SM^2^ | 60.9±12.1 | 15.0±4.7 | 14.8±4.4 | 0.2±0.9 | 1.6±1.6 | 3.9±1.6 | 49.7±3.4 |
|  | 4. Entisols, Ultisols/ Low non-Pinus BA/ Low SM | 54.7±7.5 | 13.5±2.9 | 13.2±2.7 | 0.2±0.6 | 1.2±1.0 | 5.6±1.0 | 38.5±2.1 |
|  | Reference | 25.8±9.5 | 13.1±3.8 | 12.5±3.7 | 0.6±0.5 | 0.5±1.3 | 8.0±1.4 | 42.1±3.5 |
| Savannah River Site |  |  |  |  |  |  |  |  |
|  | 1. High BA | 78.8±3.9 | 28.3±1.7 | 24.7±1.7 | 3.7±1.3 | 11.5±3 | 1.8±0.6 | 36.5±1.4 |
|  | 2. Low BA /Ag/Low fire | 53.5±7.0 | 16.8±2.9 | 16.6±3.1 | 0.2±2.4 | 7.2±5.2 | 2.1±1.0 | 36.3±2.6 |
|  | 3. Low BA /Ag/High fire | 56.7±6.4 | 17.0±2.7 | 17.0±2.9 | 0.0±2.2 | 0.9±4.8 | 5.5±0.9 | 33.4±2.4 |
|  | 4. Low BA /Forest | 54.7±4.3 | 14.4±1.8 | 13.4±2 | 1.0±1.5 | 3.7±3.3 | 3.7±0.7 | 38.8±1.6 |
|  | Reference | 54.5±7.5 | 13.0±3.1 | 10.2±3.3 | 2.8±2.5 | 1.8±5.8 | 4.6±1.2 | 39.7±2.7 |

^1^ Basal area.

^2^ Soil moisture holding capacity.
